# Domain-specific functions of Stardust in *Drosophila* embryonic development

**DOI:** 10.1098/rsos.160776

**Published:** 2016-11-16

**Authors:** Leonie Koch, Sabine Feicht, Rui Sun, Arnab Sen, Michael P. Krahn

**Affiliations:** Molecular and Cellular Anatomy, University of Regensburg, Universitätsstr. 31, 93053 Regensburg, Germany

**Keywords:** Crumbs, Stardust, PAR-6, *Drosophila*, epithelial polarity

## Abstract

In *Drosophila*, the adaptor protein Stardust is essential for the stabilization of the polarity determinant Crumbs in various epithelial tissues, including the embryonic epidermis, the follicular epithelium and photoreceptor cells of the compound eye. In turn, Stardust recruits another adaptor protein, PATJ, to the subapical region to support adherens junction formation and morphogenetic events. Moreover, Stardust binds to Lin-7, which is dispensable in epithelial cells but functions in postsynaptic vesicle fusion. Finally, Stardust has been reported to bind directly to PAR-6, thereby linking the Crumbs–Stardust–PATJ complex to the PAR-6/aPKC complex. PAR-6 and aPKC are also capable of directly binding Bazooka (the *Drosophila* homologue of PAR-3) to form the PAR/aPKC complex, which is essential for apical–basal polarity and cell–cell contact formation in most epithelia. However, little is known about the physiological relevance of these interactions in the embryonic epidermis of *Drosophila in vivo*. Thus, we performed a structure–function analysis of the annotated domains with GFP-tagged Stardust and evaluated the localization and function of the mutant proteins in epithelial cells of the embryonic epidermis. The data presented here confirm a crucial role of the PDZ domain in binding Crumbs and recruiting the protein to the subapical region. However, the isolated PDZ domain is not capable of being recruited to the cortex, and the SH3 domain is essential to support the binding to Crumbs. Notably, the conserved N-terminal regions (ECR1 and ECR2) are not crucial for epithelial polarity. Finally, the GUK domain plays an important role for the protein's function, which is not directly linked to Crumbs stabilization, and the L27N domain is essential for epithelial polarization independently of recruiting PATJ.

## 1. Introduction

Apical–basal polarity is one of the most important characteristics of many epithelial cells to accomplish their function. In these cells, the apical plasma membrane domain faces an organ lumen or the outer surface of an organism on one side, and the basal plasma membrane domain connects the epithelial cells with the basal membrane and connective tissue on the other side. Furthermore, apical–basal polarity is closely linked to the formation of cell–cell contacts, thus ensuring mechanical integrity of the tissue as well as regulation of a paracellular diffusion barrier.

Many polarity determinants, which regulate apical–basal polarity and junction assembly, are highly conserved throughout evolution, from worm to human. According to their localization and function, polarity proteins can be classified as apical polarity regulators (APRs) or basolateral polarity regulators (BLPRs) [1].

Apical and basolateral polarity proteins interact in an antagonistic way, using negative feedback mechanisms. Thus, they ensure the differentiation of the plasma membrane in an apical and a basolateral domain, which is the prerequisite for correct sorting, e.g. of channels, receptors, enzymes or lipids.

In *Drosophila*, two apical protein complexes determine the apical plasma membrane domain—partly in redundancy, depending on the tissue and developmental context: the PAR complex and the Crumbs complex. The PAR complex is composed of the serine-threonine-kinase aPKC (atypical protein kinase C), the scaffold proteins PAR-6 and Bazooka (Baz) and the GTPase Cdc42 [2,3].

The second apical polarity complex, the Crumbs complex, consists in its canonical form of the transmembrane protein Crumbs (Crb), the adaptor protein Stardust (Sdt; protein associated with Lin-7 One, Pals1 in mammals), the PDZ domain containing protein PATJ (Pals1-associated tight junction protein) and Lin-7 [4].

Crb is a key determinant of apical identity: loss of Crb in the embryonic epidermis results in a strong reduction of the apical domain (reflected by an impaired secretion of the cuticle) and a weakening of the adherens junctions (AJ) [5,6], and vice versa, overexpression of Crb enlarges the apical plasma membrane at the expense of the basolateral domain [7]. Notably, loss of Crb is at least partly compensated by simultaneous reduction of one of the basolateral polarity cues (Lethal (2) Giant Larvae, Lgl, Discs Large or Scribble) [8].

The big extracellular domain of Crb has been reported to facilitate a homophilic interaction [9], although it might be dispensable for determining apical identity [7,9,10]. By contrast, the short—highly conserved—intracellular tail contains two important protein-interaction domains: the C-terminal ERLI-motif facilitates binding to Sdt which is in turn necessary to stabilize Crb in the membrane at the subapical region [11,12]. Furthermore, PAR-6 competes with Sdt for binding the ERLI-motif of Crb *in vitro* [13,14]. Secondly, Crb is linked via the FERM (Protein 4.1—Ezrin/Radixin/Moesin) domain to the subcortical Actin cytoskeleton through the FERM domain protein Moesin and β_heavy_-spectrin [15,16]. Another function of the FERM domain is binding to Yurt, which restricts Crb activity [17]. Apart from the control of apical–basal polarity, Crb controls cell proliferation and organ growth by influencing the Hippo pathway via binding to the FERM domain protein Expanded [18–20] and by restricting the activation of Notch [21].

As Sdt stabilizes Crb at the correct subcellular position, *sdt* alleles exhibit similar phenotypes to *crb* mutants regarding apical–basal polarity [11,12,22,23].

**Figure 1.**
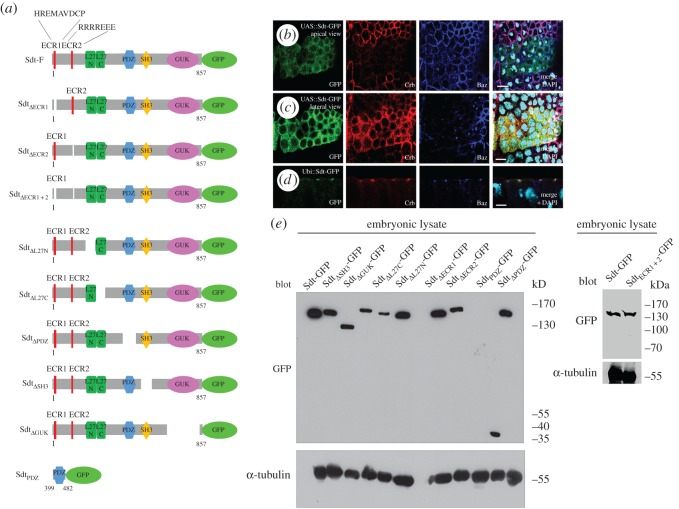
Structure–function analysis of *Drosophila* Stardust in epithelial cells. (*a*) Schematic drawing of different Sdt variants analysed in this study. The amino acid sequence of ECR1 and ECR2 is indicated in wild-type Sdt. (*b,c*) Overexpression of UAS::Sdt-GFP using en::GAL4 results in cytoplasmic Sdt-GFP localization and depletion of Crb and partially of Baz from the subapical region (focal plane in (*b*)) and an accumulation of the proteins in the cytoplasm (more lateral focal plane in (*c*)). (*d*) Ubi::Sdt-GFP localizes correctly to the apical junctions. (*e*) Western blotting of embryonic lysates expressing the Sdt variants described in this study. Pictures show embryonic stages 11–12. Scale bars, 5 µm.

However, Sdt is a large scaffold protein exhibiting several protein-interaction domains (figure 1*a*): The N-terminal evolutionary conserved regions (ECR) 1 and 2 have been reported to be involved in the binding of mammalian Pals1 to two different PAR-6 proteins in cultured mammalian cells [24–26]. However, apart from the function of a large N-terminal fragment, including ECR1/2, in apical recruitment of PAR-6 in photoreceptor cells [27], no function of this interaction in epithelia *in vivo* has been reported until now.

The first L27 (Lin-2/Lin-7) domain (L27N) is essential to recruit PATJ to the subapical region [28,29], which in turn stabilizes the AJ by promoting Myosin activity [30] but is not essential to stabilize the Crb-Sdt complex in the embryonic epidermis [28,30,31]. Via its second L27 domain (L27C), Sdt recruits Lin-7 (Veli) to the plasma membrane, which is not crucial for epithelial polarity in *Drosophila* but functions at the postsynaptic synapse membrane to prevent light-induced photoreceptor degeneration [32,33]. The PDZ domain of Sdt binds not only to the ERLI-motif of Crb but also to a conserved region of Baz [34], thus establishing two different polarity complexes which are present in parallel in the embryonic epidermis throughout embryogenesis: the Crb-Sdt-PATJ and the Baz-Sdt-PATJ complexes [29].

Apart from the PDZ domain, recent data from the crystallization of parts of the Crb-Pals1 complex reveal an important role of the SH3 (Src-homology-3) and GUK (guanylate kinase) domains in stabilizing the Crb-Pals1 complex, thus regulating lumen formation in mammalian cysts models [35].

Because limited information is available about the function of the protein-interaction domains of Sdt *in vivo*, we performed a structure–function analysis of Sdt in the embryonic epidermis of *Drosophila*.

## 2. Material and methods

### 2.1. Plasmids

The ORF of Sdt-F (formerly described as Sdt-B1) was cloned into pENTR (Life Technologies). Deletions of the distinct domains (introducing a flexible 3xGlycin spacer instead of the domain) were established by mutagenesis PCR using the following oligonucleotides:

SdtDECR1-F: 5′-CAAGATAACGGTCCAGGTGGAGGTGACACGTTCATCGCA-3′—deletion of aa11-19;
SdtDECR2-F: 5′-TACCAGGAGCAACTGGGAGGTGGAGAGCGCATAGCGCAG-3′—deletion of aa119-125;
SdtDL27N-F: 5′-GCGGAACAGATCGATGGTGGAGGTTCTGGTCCACTGCAT-3′—deletion of aa193-250;
SdtDL27C-F: 5′-CGCGTCTCTGGTCCAGGAGGTGGAGGCACGCCCTCGCCA-3′—deletion of aa254-304;
SdtDPDZ-F: 5′-ATCATCCAGATCGAGGGAGGTGGACCAGCGGGTAGTCCA-3′—deletion of aa405-476;
SdtDSH3-F: 5′-GACACCGCCGTGTTGGGAGGTGGACAGTCGTTCCAGCAT-3′—deletion of aa509-568;
SdtDGUK-F: 5′-GCTACCCACAAGCGGGGAGGTGGACAATGGGTGCCCGCC-3′—deletion of aa657-841.

The ORF of Sdt variants was subcloned into UWGattB (modified from UWG, which was obtained from the Drosophila Genomic Resource Center as described before [29]) vector by clonase reaction (Life Technologies).

### 2.2. Fly stocks and genetics

Fly stocks were cultured on standard cornmeal agar food and maintained at 25°C. Transgenic flies of UAS::Sdt-GFP and the Ubi::Sdt-GFP variants were established using the Phi-C31-Integrase system [36] with attP40 (for the UAS construct) and attPVK00037 (22A, for the Ubi::Sdt-GFP constructs). En::GAL4 and arm::GAL4 were obtained from the Bloomington stock center. The *sdt^K85^* allele [37] was used for evaluation of the function of (mutant) Sdt proteins *in vivo* by producing germ line clones with the female sterile OvoD technique [38] using FRT19A-OvoD1, hs::Flp (BL#23880). Females of *sdt^K85^*, FRT19A-OvoD1, FRT19A; Ubi::Sdt-GFP were mated with males carrying an FM7-ChFP-fluorescent balancer [39] and Ubi::Sdt-GFP. Embryos which were homozygous for *sdt^K85^* were identified using staining against sex lethal [40] for immunostainings and by sorting against ChFP for cuticle preparations and lethality tests. The *crb^11A22^* allele [41] was used for cuticle preparations of *crb*-mutant embryos. For lethality tests, *sdt^K85^*-mutant embryos expressing the Sdt protein variants were generated as described above. In three independent experiments, 100 homozygous mutant embryos were scored (in each experiment) for embryonic lethality, L1/L2, L3 and pupal lethality and surviving flies. Error bars indicate the standard error of the mean.

#### 2.2.1. Generation of an antibody against Sdt

An antibody directed against the PDZ domain of Sdt was raised by immunizing rabbits with GST-Sdt_PDZ_ (Davids Biotechnology, Regensburg, Germany). The specificity of the serum was tested in immunostainings on *sdt^K85^* mutant embryos (electronic supplementary material, figure S1*c*,*d*).

#### 2.2.2. Immunoprecipitation and western blotting

For immunoprecipitations, w^-^ embryos or embryos expressing GFP-tagged Sdt variants from an overnight collection were dechorionated and lysed in lysis buffer (1% Triton X-100, 150 mM NaCl, 1 mM CaCl_2_, 1 mM MgCl_2_, 50 mM Tris-HCl pH 7.5) supplemented with protease inhibitors. After centrifugation, either mouse anti Sdt antibody [42], mouse anti β-galactosidase or rabbit anti GFP (Life Technologies) was added to embryonic lysate corresponding to 500 µg total protein. Immune complexes were harvested using protein A/G-conjugated agarose (BioVision). Beads were washed five times in lysis buffer and boiled in 2× SDS sample buffer before SDS-PAGE and western blot. Immunoprecipitation from Schneider 2R cells transfected with Sdt-GFP (Sdt UWGattB) and Ubi::PAR-6-myc (PAR-6 UWM) was similarly performed as described previously [30]. Western blotting was done according to standard procedures. The primary antibodies used for western blotting were as follows: mouse anti Crb (Cq4, 1 : 50, DSHB), mouse anti Sdt (1 : 20, [42]), guinea pig anti PATJ (1 : 1000, [30]), rabbit anti Baz (1 : 2000, [43]), rabbit anti aPKC (aPKCζ, 1 : 500, Santa Cruz sc-216), mouse anti GFP (1 : 500, B2, Santa Cruz sc-9996) and guinea pig anti PAR6 (1 : 500, [44]).

#### 2.2.3. Immunohistochemistry

Embryos were fixed in 4% formaldehyde, phosphate buffer pH 7.4 as previously described [45]. The primary antibodies used for indirect immunofluorescence were as follows: guinea pig anti PATJ (1 : 500, [30]), mouse anti Sdt (1 : 20, [42]), rabbit anti Sdt (1 : 2000, this study), rabbit anti Baz (1 : 1000, [43]), mouse anti Crb (Cq4, 1 : 50, DSHB), mouse anti Dlg (1 : 50, DSHB), rabbit anti GFP (#A11122, 1 : 1000, Life Technologies) and chicken anti GFP (1 : 2000, Aves Laboratories). Secondary antibodies conjugated with Alexa 488, Alexa 568 and Alexa 647 (Life Technologies) were used at 1 : 400.

Images were taken on a Zeiss LSM 710 Meta confocal microscope and processed using Adobe Photoshop.

## 3. Results and discussion

### 3.1. Sdt exhibits several evolutionary conserved protein-interaction domains

Similar to other MAGUK (membrane-associated GUK) proteins, e.g. discs large, ZO-1, calcium/calmodulin-dependent serine protein kinase or other members of the MPP (MAGUK p55 subfamily) family, Sdt contains a GUK domain, which is catalytically inactive but might facilitate protein–protein interaction, as well as a single PDZ domain (binding to Crb and Baz [11,12,34,46]), an SH3 domain and two L27 domains (L27N facilitating assembly of the Sdt-PATJ complex and L27C recruiting Lin-7/Veli [29,32,46–48]).

Furthermore, two highly conserved short amino acid motifs (ECR1 = HREMAVDCP and ECR2 = RRRREEE, figure 1*a*) have been identified in mammalian Pals1 to mediate an interaction with the PDZ domain of PAR-6 [24–26]. Interestingly, this interaction is only seen in Sdt isoforms, which do not exhibit a long spacer between ECR1 and ECR2 due to alternative splicing (formerly annotated as Sdt-B1 [11,27,37]).

Some insights into the *in vivo* relevance of the described protein interactions have already been drawn from structure–function analyses in photoreceptor cells of the developing *Drosophila* eye [27], a study in zebrafish using alleles and morpholino-knock-down of Nagie oko, the zebrafish homologue of Sdt [49], and expression of Pals1 variants in cultured mammalian epithelial cells in a wild-type background [46].

In order to investigate the relevance of the protein-interaction domains for epithelial polarization in the *Drosophila* epidermis and for development of the fly, we performed a structure–function analysis of diverse Sdt variants (as depicted in figure 1*a*) in a wild-type background as well as in an *sdt*-mutant background. Even mild overexpression of Sdt (using the UAS/GAL4 system with a weak *armadillo* promoter or *engrailed* promoter at 18°) causes a strong cytoplasmic mislocalization of the overexpressed protein as well as of Crb, polarity defects and embryonic lethality (figure 1*b*,*c* and data not shown). To avoid these overexpression artefacts, we expressed Sdt from a ubiquitous promoter (*ubiquitin*, *ubi*), resulting in a physiological localization at subapical region (figure 1*d*) and a robust rescue capacity (figure 4*a*). We decided to focus on the Sdt-F isoform (formerly annotated as Sdt-B1 [11,12,37,42]) because this isoform has been reported to be expressed throughout embryonic development and to be capable of interacting with PAR-6 as outlined above. We confirmed by western blotting the expression of a band around 110 kDa (predicted size of Sdt-F: 94 kDa), which probably corresponds to Sdt-F, from gastrulation onwards (electronic supplementary material, figure S1*b*). In addition, a second specific band appeared slightly above, which either corresponds to a newly identified isoform (Sdt-D [42]) or represents protein modifications of Sdt-F. Notably, Sdt-B (previously annotated as Sdt-MAGUK1 [11] or Sdt-A [37], predicted size 139 kDa) is only expressed at very low levels at the end of embryogenesis (electronic supplementary material, figure S1*b*, asterisk).

Although expression of Sdt-F in an *sdt*-mutant background results in a rescue of around 40% hatched flies and suppression of the apical–basal polarity phenotype in the embryonic epidermis, still approximately 40% of the rescued embryos die at the end of embryogenesis (or fail to hatch as larvae), showing no obvious polarity defects (figures 3*b* and 4*d*,*m*). This might be either due to the artificial promoter, which expresses Sdt in all tissues and might produce dominant negative effects, or due to the lack of further isoforms, which might be essential in a distinct developmental context [37,42]. However, with respect to apical–basal polarity of the embryonic epidermis, analyses of *sdt*-mutant embryos rescued with Sdt-F show a full rescue capacity and no polarity defects (figure 3*b* and data not shown). All Sdt variants were expressed with a C-terminal GFP and can be detected at the correct size in western blotting (figure 1*e*).

### 3.2. The PDZ and SH3 domain of Sdt cooperate to stabilize the Crb–Sdt complex

**Figure 2.**
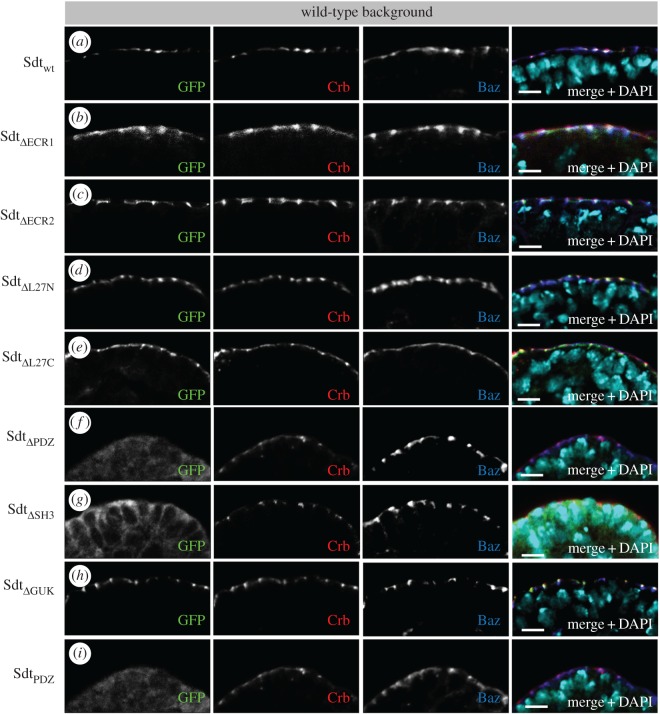
Localization of Sdt variants in wild-type epithelial cells. (*a–e*,*h*) Deletion of the ECR1, ECR2 motif or the L27N, L27C or GUK domain does not impair localization of the mutant protein at the subapical region of wild-type epithelial cells, where it colocalizes with Crb and Baz. (*f*) Sdt protein, which lacks the PDZ domain, results in a rather cytoplasmic localization of the mutant protein with some residual staining at the lateral membrane. (*g*) The SH3 domain supports correct targeting to the subapical region and Sdt_ΔSH3_ mislocalizes—similar to Sdt_ΔPDZ_—to the cytoplasm and to the lateral cortex. (*i*) The isolated PDZ domain fused to GFP is not sufficient to localize to the (apical) junctions. All Sdt proteins were expressed with a ubiquitous promoter (Ubi::Sdt-GFP) from two chromosomes carrying the insertion. Pictures show embryonic stages 11–12. Scale bars, 5 µm.

First, we investigated which domains are necessary to target the protein to the subapical region. In agreement with previous reports, the PDZ domain, which recruits Sdt either to Crb or to Baz at the subapical region [11,12,34,46,49], is essential for correct subcellular localization of Sdt. Deletion of the PDZ domain results in a strongly disturbed and mostly cytoplasmic localization of the mutant protein (figure 2*f*), whereas wild-type Sdt-GFP correctly localizes to the subapical region (figures 1*d* and 2*a*).

**Figure 3.**
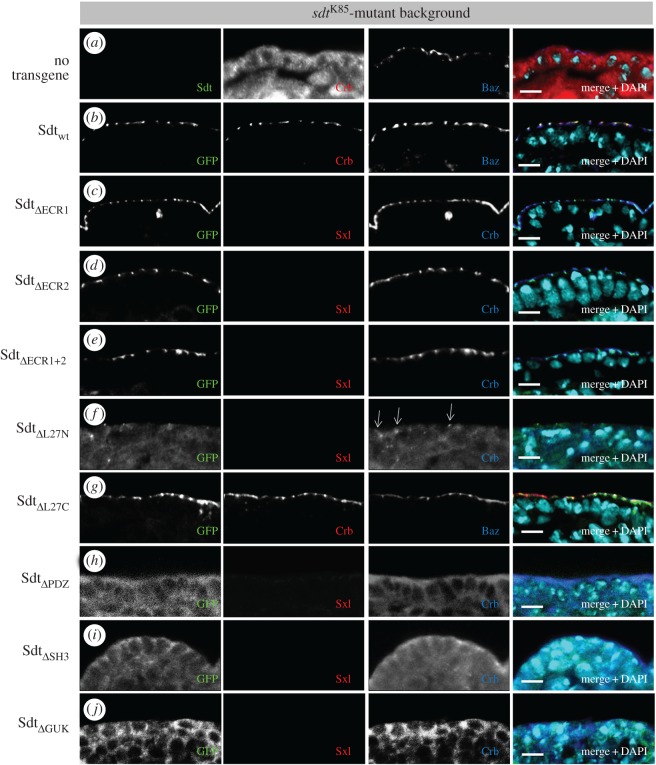
Several protein–protein interaction domains of Sdt are essential for epithelial cell polarity and fly development. (*a*–*e*,*g*) Expression of wild-type Sdt, Sdt_ΔECR1_, Sdt_ΔECR2_, Sdt_ΔECR1+2_ or Sdt_ΔL27C_ in *sdt*-mutant cells of the embryonic epidermis rescues apical–basal polarity and Crb localization at the subapical region. (*f*,*h–j*) Expression of Sdt_ΔL27N_, Sdt_ΔPDZ_, Sdt_ΔSH3_ or Sdt_ΔGUK_ in *sdt*-mutant embryos results in disturbed apical–basal polarity and mislocalization of Crb. *sdt^K85^*-mutant embryos were either derived from homozygous parents (Sdt_wt_, Sdt_ΔL27C_) or germ line clones were generated as described in Material and methods and hemizygous mutant embryos were identified using staining against sex lethal. All Sdt proteins were expressed with a ubiquitous promoter (Ubi::Sdt-GFP) from two chromosomes carrying the insertion. Pictures show embryonic stages 11–12. Scale bars, 5 µm.

**Figure 4.**
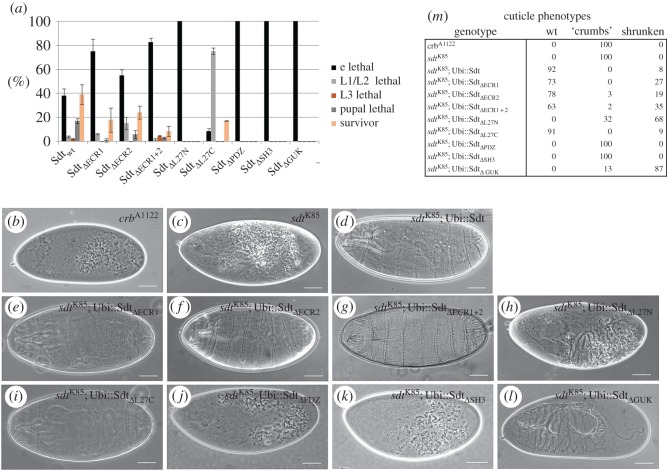
Functional analysis of Sdt domains in development of *Drosophila*. (*a*) Lethality test of different Sdt variants used in this study. For each of the three experiments, 100 homozygous mutant embryos derived from *sdt^K85^* germ line clones were identified using the FM7-ChFP balancer. (*b*,*c*) Embryos homozygous mutant for *crb* or *sdt* die during embryonic development and fail to secret more than debris of cuticle (‘crumbs’ phenotype), whereas expression of Sdt-GFP rescues this defect to a normal cuticle in most embryos (*d*,*m*). The majority of *sdt*-mutant embryos rescued by expressing Sdt_ΔECR1_ (*e*,*m*), Sdt_ΔECR2_ (*f*,*m*), Sdt_ΔECR1+2_ (*g*,*m*) or Sdt_ΔL27C_ (*i*,*m*) exhibit a regular cuticle. In embryos with Sdt_ΔL27N_-rescue, one-third of the embryos exhibit a ‘crumbs’ phenotype, whereas in two-thirds of the embryos the cuticle is shrunken, shows holes and lacks head structures (*h*,*m*). Embryos with Sdt_ΔPDZ_-rescue (*j*,*m*) or Sdt_ΔSH3_-rescue (*k*,*m*) phenocopy the cuticle phenotype of *sdt*. (*l*,*m*) Rescue of *sdt* with Sdt_ΔGUK_ produces a shrunken cuticle in most cases with holes and defects in head development. In total, 100 cuticles were scored for each genotype. Scale bars, 200 µm.

**Figure 5.**
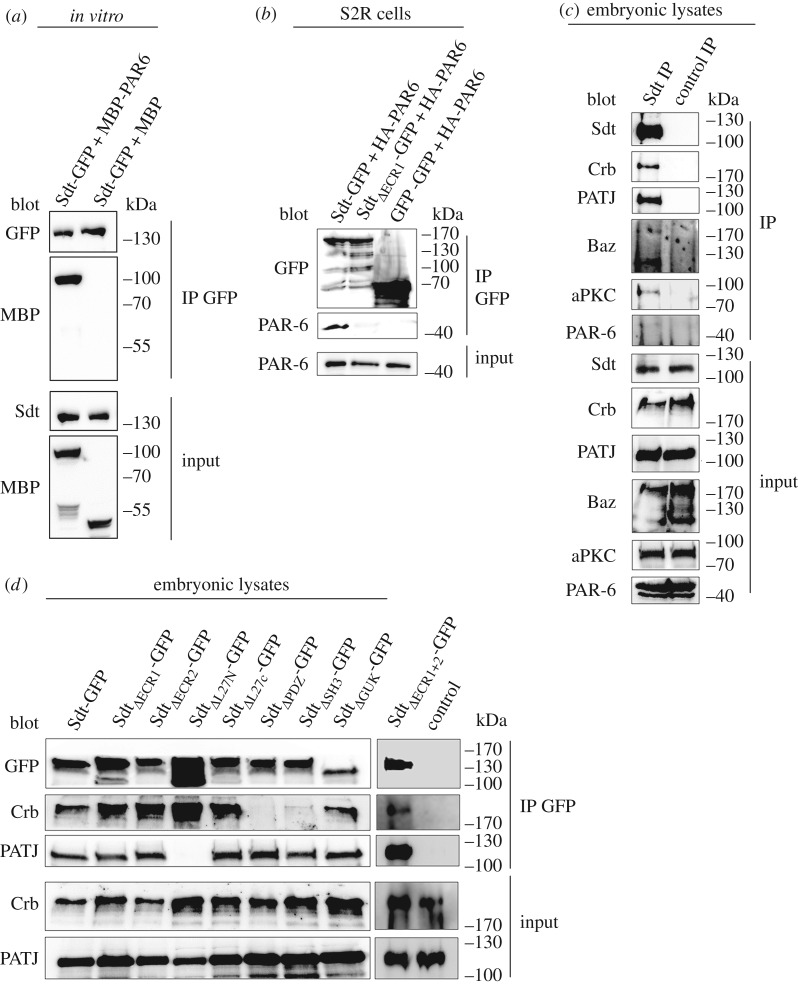
Association of Sdt with different polarity proteins. (*a*) Recombinant MBP-PAR-6 but not MBP alone interacts directly with Sdt-GFP, which was purified from transfected S2R cells. (*b*) Overexpressed HA-PAR-6 co-immunoprecipitates with Sdt-GFP but not with Sdt_ΔECR1_ or 2xGFP (negative control). (*c*) Endogenous Crb, PATJ, Baz and aPKC but not PAR-6 co-immunoprecipitate with endogenous Sdt from embryonic lysates. Control IP = IP with anti-β-galactosidase antibody. (*d*) Sdt variants were immunoprecipitated using an anti GFP antibody, and proteins bound to the chimeric proteins were identified by western blotting. All Sdt proteins were expressed with a ubiquitous promoter (Ubi::Sdt-GFP) from two chromosomes carrying the insertion.

Strikingly, the PDZ domain alone is not sufficient to accomplish cortical recruitment (figure 2*i*), although the small PDZ-GFP chimeric protein is stable (figure 1*e*). A recent study suggests that apart from the PDZ domain, the SH3 and GUK domains play a crucial role to support the formation and stability of the Crb–Pals1 complex [35]. Indeed deletion of the SH3 domain results in a rather cytoplasmic localization of the mutant protein (figure 2*g*), although some protein remains at the cell membrane but exhibits a broader localization along the lateral membrane. Although Sdt_ΔPDZ_ or Sdt_ΔSH3_ are still capable of binding endogenous PATJ (figure 5*d*), PATJ is not displaced from the subapical region by the cytoplasmic Sdt variants (electronic supplementary material, figure S2*b*,*c*). Co-immunoprecipitation experiments of mutant Sdt proteins with endogenous Crb demonstrate that apart from the PDZ domain, the SH3 domain of Sdt is indeed essential for the association of Sdt with Crb (figure 5*d*). Consequently, the expression of Sdt_ΔPDZ_ or Sdt_ΔSH3_ does not rescue the cuticle phenotype of *sdt^K85^* (figure 4*c*,*d*,*j,k*) or Crb localization/apical–basal polarity in epithelial cells of the embryonic epidermis (figure 3*h*,*i*). These results are in line with previous studies in zebrafish retinal neural and pigmented epithelia and *Drosophila* photoreceptor cells describing a crucial role of the PDZ domain [27,49] and the SH3 domain [27] in these apical–basal polarized cell types. By contrast, deletion of the SH3 domain does not affect targeting of Pals1 to the tight junctions (TJ) in cultured mammalian cells [46].

By contrast, deletion of the GUK domain does not affect localization of the mutant protein in wild-type epithelial cells or its association with Crb (figures 2*h* and 5*d*).

### 3.3. The ECR domains are not essential for apical–basal polarity in the embryonic epidermis

Apart from the canonical Crb–Sdt–PATJ complex, several interactions between the PAR/aPKC and the Crb complex have been suggested: endogenous Sdt and PATJ associate with Baz (but not in a quarternary complex Crb–Sdt–PATJ–Baz), probably by direct binding of Sdt to Baz [29,34]. aPKC binds to and phosphorylates Crb *in vitro* [50], although this phosphorylation is not essential for epithelial polarity and fly development [51] (R.S. and M.P.K. 2016, unpublished data). PAR-6 binds directly to the ERLI-motif of Crb [13,14] as well as to the ECR1/ECR2 domains of Pals1 [24–26] *in vitro* and under overexpression conditions in mammalian cultured cells. An *in vivo* relevance of the Sdt–PAR-6 interaction might be deduced from the mislocalization of PAR-6 in *sdt*-mutant photoreceptor cells, which are rescued by Sdt variant lacking the entire N-terminus including the ECR-motifs and the L27N domain [27]. We found that Sdt_ΔECR1_ and Sdt_ΔECR2_ localize correctly at the subapical region in the embryonic epidermis (figure 2*b*,*c*). Furthermore, expression of these transgenes as well as the double mutant protein (Sdt_ΔECR1+2_) in an *sdt*-mutant background restores epithelial polarity and Crb localization (figure 3*c*–*e*). However, lethality tests reveal a higher embryonic lethality of *sdt*-mutants expressing either Sdt_ΔECR1_ or Sdt_ΔECR2_ compared with wild-type Sdt, with an even more increased lethality phenotype for Sdt_ΔECR1+2_ (figure 4*a*), although immunostainings with polarity markers (figure 3*c*–*e* and data not shown) do not reveal obvious polarity defects. Furthermore, cuticle preparations demonstrate secretion defects in only a minority of embryos (figure 4*e*–*g*,*m*). Hatched flies of these genotypes appear normal (data not shown). We confirmed that Sdt and PAR-6 can interact *in vitro* and under overexpression conditions in Schneider-2R cells (S2R cells) and that this interaction depends on ECR1 (figure 5*a*,*b*). However, upon immunoprecipitation of endogenous Sdt from wild-type embryos, we were not able to detect substantial amounts of PAR-6 to associate with Sdt whereas Crb, PATJ, Baz and aPKC co-immunoprecipitated with Sdt (figure 5*c*). Moreover, mutation of either ECR1 or ECR2 (or both together) does not impair the assembly of the Crb–Sdt complex *in vivo* (figure 5*d*).

These data suggest that the ECR1 and ECR2 motifs are not essential for a robust binding (which can be detected by co-immunoprecipitation) of Sdt with PAR-6 or a stabilization of the Crb–Sdt–PATJ complex under endogenous conditions and are dispensable for apical–basal polarity but are to some extent crucial for efficient embryonic development.

### 3.4. The L27N domain exhibits functions beyond binding of PATJ

We recently revealed that the association of PATJ with both apical polarity complexes (the Crb and the Baz complexes) is essential for its function [29]. The recruitment to these polarity landmarks is facilitated by the L27N domain of Sdt, which heterodimerizes with the L27 domain of PATJ [47]. Consequently, deletion of the L27 domain in PATJ abolishes binding to Sdt and localization of the mutant protein at the subapical region [28,29]; vice versa, deletion of L27N in Sdt does not affect the protein's localization in wild-type epithelial cells (figure 2*d*) but disturbs binding to PATJ (figure 5*d*). We and others revealed that PATJ is not crucial for apical–basal polarity or stabilization of the Crb–Sdt complex in the embryonic epidermis [28,30,31] but *PATJ*-mutant flies die during early puparation, showing no obvious metamorphosis, which is—at least partly—due to a decreased Myosin activation [30]. Surprisingly, *sdt*-mutant flies expressing the Sdt_ΔL27N_ transgene show a complete embryonic lethality (figure 4*a*), exhibiting strong polarity and cuticle defects with some residual intact cuticle left, which might be due to some apically localized Crb protein (figure 3*f*, arrows; and figure 4*h*).

A possible explanation for this finding is that L27N facilitates the formation of a stable supramolecular Crb–Sdt(–PATJ) complex by either hetero-oligomerization with PATJ or homo-oligomerization with another Sdt molecule. However, the first possibility can be ruled out because loss of PATJ does not affect the stability of Crb–Sdt complex in the embryonic epidermis [28,30,31] and embryos which are homozygous mutant for *PATJ* neither show polarity defects in the embryonic epidermis nor exhibit a fully penetrant embryonic lethality [30]. The second possibility is very unlikely because first homo-oligomerization of Sdt/Pals1 seems to be biochemically unfavourable [47] and second Sdt_ΔL27N_ robustly associates with endogenous Crb (figure 5*d*). A third explanation would be that unbound PATJ (which does not associate with Sdt anymore) exhibits a dominant negative effect (as overexpression of PATJ does [30]). However, we did not observe a rescue effect of *sdt-PATJ* double mutants expressing SdtΔL27N, which would support this hypothesis (data not shown).

Thus, the L27N domain might accomplish more crucial functions during epithelial polarization of the embryonic epidermis apart from recruiting PATJ.

In contrast with the L27N domain, the L27C domain does not seem to be essential for epithelial polarization and fly development because SdtΔL27C can rescue the polarity phenotypes of *sdt^K85^* and produces surviving flies (figures 3*g* and 4*a*,*i*). This is in line with previous results that identify Lin-7/Veli as an interaction partner of SdtL27C and describe a role for Lin-7/Veli in postsynaptic signal transmission but not for epithelial polarity or fly development [32,33]. The increased early larval lethality might indeed be due to defects in synapse formation. Although this is in contrast with findings in mammalian cells, which suggest a supportive role of Veli in stabilization of the Crb–Pals1 complex [48], we could not detect a decreased targeting of apical-junctional Sdt_ΔL27C_ or Crb or a weaker association of mutant Sdt with Crb (figure 5*d*).

### 3.5. The GUK domain is not essential for stabilization of the Crb–Sdt complex but necessary for embryonic development

As mentioned above, deletion of the GUK domain affects neither localization of Sdt to the subapical region in wild-type cells nor its association with Crb (figures 2*h* and 5*d*). However, in an *sdt^K85^*-mutant background, expression of Sdt_ΔGUK_ does not rescue the apical localization of Crb or the embryonic lethality (figures 4*a* and 3*j*). Epithelial polarity and embryonic morphology are severely disrupted, which is reflected by a disturbed cuticle secretion: cuticles from *sdt^K85^*; Sdt_ΔGUK_ embryos are shrunken and display big holes in the dorsal cuticle (figure 4*l*). This is different from *sdt^K85^* cuticles which exhibit only crumbles of cuticle and thus phenocopies a *crb* loss of function allele (figure 4*b*,*c*). In contrast with *sdt^K85^*-mutant embryos (figure 3*a*), in which Crb is cytoplasmic/vesicular, *sdt^K85^*; Sdt_ΔGUK_ embryos exhibit to some extent a cortical localization, although randomly distributed (figure 3*j*). However, this residual cortical localization seems to be sufficient to produce a segmented, although shrunken and irregular cuticle (figure 4*l*).

Therefore, we cannot verify a role for the GUK domain of Sdt in stabilizing the Crb–Sdt complex *in vivo*. Nonetheless, the GUK domain is essential for the function of Sdt during epithelial polarization, which might be independent of its canonical role to stabilize Crb.

Taken together our findings confirm an essential role of the PDZ-SH3 domain tandem in binding and stabilization of Crb *in vivo*. By contrast, we did not confirm a function of ECR1 and ECR2 in epithelial polarization *in vivo*—binding of Sdt to PAR-6 does not seem to take place at a substantial level in embryonic epithelia and, as Sdt_ΔECR1_- and Sdt_ΔECR2_-mutant embryos do not exhibit polarity phenotypes, it is unlikely that the PAR-6/Sdt interaction contributes to the establishment of apical–basal polarity but might rather have other functions, which are not related to Crb stabilization. This is in contrast with the function of these domains in photoreceptor cells, where the N-terminus of Sdt-H (originally described as Sdt-B2, an isoform which is exclusively expressed in heads), including ECR1, ECR2 and L27N, is important for the recruitment of PAR-6 in pupae and localization to the stalk membrane in adults [27]. However, it remains to be clarified whether this phenotype is due to loss of PAR-6 binding (upon deletion of ECR1 and ECR2) or due to impaired binding of Sdt to PATJ (deletion of the L27N domain), or both. Indeed, loss of PATJ in photoreceptor cells results in mislocalization of Crb and PAR-6 [31].

On the other hand, Sdt isoforms containing a larger stretch of amino acids between ECR1 and ECR2 (e.g. Sdt-B) are not capable of fully targeting PAR-6 in photoreceptor cells. Similar, the ECR1 and L27N domains are essential for retinal neuron organization and myocard development in zebrafish [49], whereas ECR1 seems to be dispensable for apical–basal polarity in the retinal pigmented epithelium and the overall fish morphology. This further underlines the importance of the cellular and temporal context for the function of Sdt in polarized tissues, suggesting a crucial role of the Sdt–PAR-6 interaction in neural cells (*Drosophila* photoreceptor cells and zebrafish retinal neurons) but not in other epithelia (*Drosophila* epidermis, retinal pigmented epithelium).

Moreover, our data indicate a new, PATJ-independent function of the L27N domain, which is in line with previous reports demonstrating that mutant Nagie oko protein, which cannot bind PATJ fails to rescue body form defects and the neural retinal phenotype of a Nagie oko mutant allele in zebrafish [49]. Deletion of the L27N domain does not affect localization of the mutant protein in the epidermis of wild-type *Drosophila* (which is in agreement with the observation that deletion of PATJ does not affect Sdt localization [30]), whereas this domain seems to be crucial for TJ targeting of Pals1 in cultured mammalian cells [46].

Finally, we demonstrate that the GUK domain of Sdt is crucial for apical–basal polarity of the embryonic epidermis and for embryonic development. Similar, deletion of the GUK domain is indispensable for photoreceptor polarity in the adult *Drosophila* eye [27]. However, we demonstrate that the GUK domain is neither essential for stabilizing binding to Crumbs as suggested recently [35] nor important for the localization of the protein in wild-type epithelial cells. These data hint to an essential, Crb-independent role of the GUK domain during epithelial polarization and embryonic development which was not addressed in previous studies [27,46,49].

## Supplementary Material

Figure S1. Expression of Sdt in embryogenesis. (A) Extracts of 0-16h old embryos of either sdtK85 germ lines clones (mated with males carrying an FM7-ChFP balancer and sorted against ChFP) or wild type flies were blotted against Sdt and Actin. Both bands seen above the 100kDa marker band appear to be specific. (B) Extracts of staged embryos were blotted against Sdt and Actin. (C-D) Heterozygous and zygotic mutant sdtK85 embryos were stained with anti Sdt and DAPI. Note that the morphology of sdtK85 mutant embryos is already disturbed. Scale bars = 50μm.

## Supplementary Material

Figure S2. Mislocalized Sdt variants do not displace endogenous PATJ or PAR-6. (A-D) Sdt-GFP was ubiquitous expressed in wild type embryos and distribution of GFP, PATJ and PAR-6 was analyzed in stage 11-12 embryos. Scale bars = 5μm.

## Data accessibility

Electronic supplementary material data are available in the electronic supplementary material at Dryad (http://dx.doi.org/10.5061/dryad.n5fr8) [52]. Supplementary figure 1 shows the expression of endogenous Sdt in embryonic lysates of different stages and supplementary figure 2 shows PATJ stainings on wild-type background with different Sdt variants.
