# Supplementary figures and images for: Domain-specific functions of Stardust in Drosophila embryonic development
Source: R Soc Open Sci. 2016 Nov 16;3(11):160776. doi: 10.1098/rsos.160776 (PMC5180163; doi:10.1098/rsos.160776)

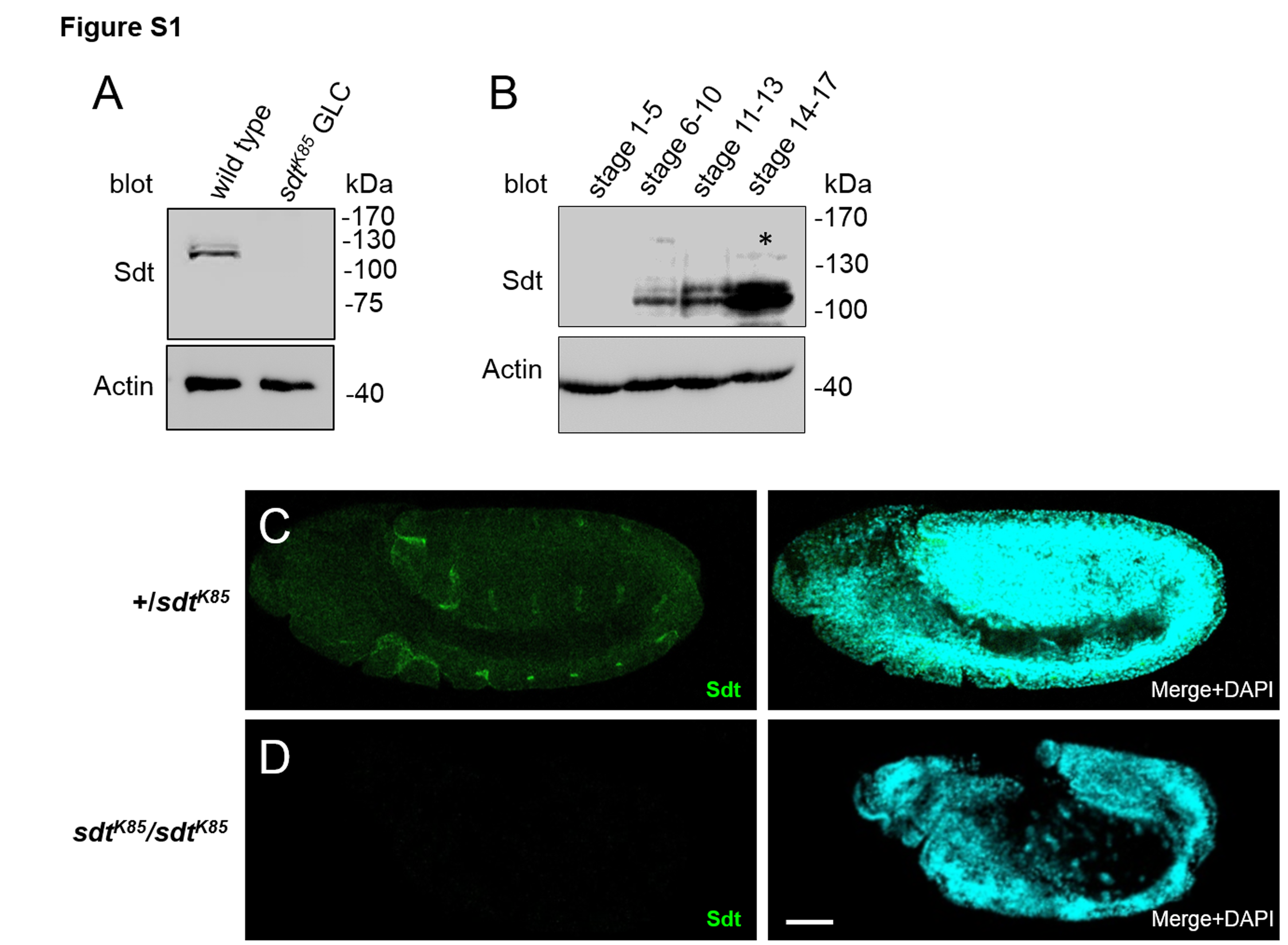

Supplement: Figure S1. Expression of Sdt in embryogenesis. (A) Extracts of 0-16h old embryos of either sdtK85 germ lines clones (mated with males carrying an FM7-ChFP balancer and sorted against ChFP) or wild type flies were blotted against Sdt and Actin. Both bands seen above the 100kDa marker band appear to b [file rsos160776supp1.tif]

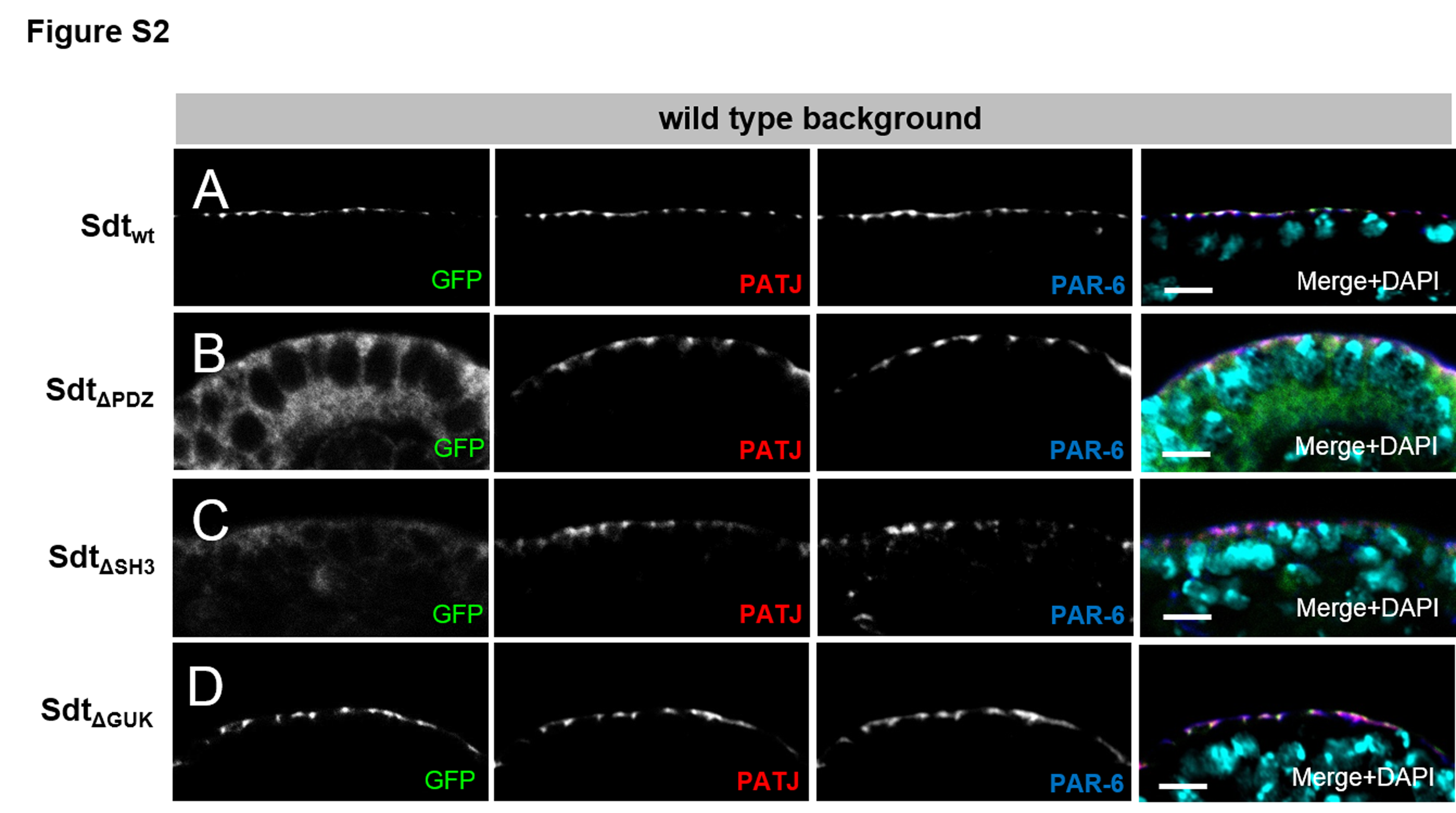

Supplement: Figure S2. Mislocalized Sdt variants do not displace endogenous PATJ or PAR-6. (A-D) Sdt-GFP was ubiquitous expressed in wild type embryos and distribution of GFP, PATJ and PAR-6 was analyzed in stage 11-12 embryos. Scale bars = 5μm. [file rsos160776supp2.tif]
